# Food Consumption, Knowledge, Attitudes, and Practices Related to Salt in Urban Areas in Five Sub-Saharan African Countries

**DOI:** 10.3390/nu10081028

**Published:** 2018-08-07

**Authors:** Magali Leyvraz, Carmelle Mizéhoun-Adissoda, Dismand Houinato, Naby Moussa Baldé, Albertino Damasceno, Bharathi Viswanathan, Mary Amyunzu-Nyamongo, Jared Owuor, Arnaud Chiolero, Pascal Bovet

**Affiliations:** 1 Institute of Social and Preventive Medicine (IUMSP), Canton University Hospital (CHUV), 1010 Lausanne, Switzerland; magali.leyvraz@chuv.ch (M.L.); arnaud.chiolero@chuv.ch (A.C.); 2 School of Nutrition and Dietetics, Faculty of Health Science, University of Abomey-Calavi, Cotonou 01 BP 526, Benin; carmelle.mizehoun@gmail.com; 3 Laboratory of Noncommunicable and Neurologic Diseases Epidemiology, Faculty of Health Science, University of Abomey-Calavi, Cotonou 01 BP 526, Benin; dshouinato@gmail.com; 4 Department of Endocrinology and Diabetes, Donka University Hospital, Conakry, Guinea; naby.balde@gmail.com or naby@afribone.net.gn; 5 Department of Medicine, Eduardo Mondlane University, Maputo, Mozambique; tino_7117@hotmail.com; 6 Ministry of Health, Victoria, Republic of Seychelles; barathi.viswanathan@health.gov.sc; 7 African Institute for Health and Development (AIHD), Nairobi 00100, Kenya; mnyamongo@aihdint.org (M.A.-N.); jowuor@aihdint.org (J.O.); 8 Institute of Primary Health Care (BIHAM), University of Bern, 3012 Bern, Switzerland

**Keywords:** salt, sodium, hypertension, knowledge, attitudes, practices, diet, Africa, Benin, Guinea, Mozambique, Kenya, Seychelles

## Abstract

High salt intake is a major risk factor of hypertension and cardiovascular disease. Improving knowledge, attitudes, and practices (KAP) related to salt intake in the general population is a key component of salt reduction strategies. The objective of this study was to describe and compare the KAP of adults related to salt in urban areas of five countries in sub-Saharan Africa. The survey included 588 participants aged 25 to 65 years who were selected using convenience samples in the urban areas of Benin, Guinea, Kenya, Mozambique, and Seychelles. Socio-demographic and food consumption were assessed using a structured closed-ended questionnaire administered by survey officers. Height, weight, and blood pressure were measured. Food consumption varied largely between countries. Processed foods high in salt, such as processed meat, cheese, pizzas, and savory snacks were consumed rather infrequently in all the countries, but salt-rich foods, such as soups or bread and salty condiments, were consumed frequently in all countries. The majority of the participants knew that high salt intake can cause health problems (85%) and thought that it is important to limit salt intake (91%). However, slightly over half (56%) of the respondents regularly tried to limit their salt intake while only 8% of the respondents thought that they consumed too much salt. Salt and salty condiments were added most of the time during cooking (92% and 64%, respectively) but rarely at the table (11%). These findings support the need for education campaigns to reduce salt added during cooking and for strategies to reduce salt content in selected manufactured foods in the region.

## 1. Introduction

Cardiovascular disease (CVD) is the leading cause of deaths worldwide including in low-income and middle-income countries (LMICs) [1]. It is estimated that high salt intake accounts for 9.5% of all CVD deaths globally due to its effect on blood pressure [2]. In sub-Saharan Africa (SSA), approximately 800,000 deaths per year are due to CVD and 6% of these deaths are attributable to high salt intake [2]. The disease burden related to elevated salt intake is expected to further increase over the next decade in LMICs [3] due to the growing and aging populations and trends towards urbanization and westernization of the diet in these countries.

Reducing dietary salt intake in the population is a key strategy to reduce the CVD burden [4]. Strategies to reduce salt intake at the population level include awareness campaigns advising individuals to reduce their salt consumption, food labeling, and reformulation of selected industrially produced foods [5]. Improving knowledge, attitudes, and practices (KAP) related to salt intake in the population is an important part of any salt reduction strategy [6]. In particular, dietary advice can reduce salt intake, blood pressure, and other CVD risk factors [7,8]. In 2015, 40 countries worldwide were implementing some organized policy or program to reduce salt consumption in the population but only one program was implemented in SSA (i.e., in South Africa) [9].

Only a few studies have examined KAP related to salt intake in SSA countries [10,11,12,13,14]. These studies have generally found low levels of KAP related to salt intake. However, most of these studies were conducted in specific population groups (e.g., hypertensive patients) and in selected settings. Given the scarcity of data on KAP related to salt intake and the high burden of CVD in LMICs [15], it was recently recommended that more studies should be conducted to assess dietary patterns and key sources of sodium in these countries [16].

The objective of this study was to describe and compare the food consumption, KAP related to salt intake, and associated factors in adults from the general population in urban areas of five SSA countries. This information is expected to be useful for guiding the development of salt reduction programs and policies in SSA.

## 2. Materials and Methods

Cross-sectional surveys were conducted in a main city in each of the five countries (Bohicon in Benin, Conakry in Guinea, Mombasa in Kenya, Maputo in Mozambique, and Victoria in Seychelles) between January 2012 and April 2013. The countries were selected based on the geographical diversity and the presence of investigators who expressed interest in examining these issues in the countries.

For four countries, participant selection was based on the convenience of a three-stage sampling strategy. The first stage was the selection of two areas in the selected cities. The second stage was the selection of households within each area and the third stage was the selection of one person within a household. The person was chosen to ensure similar numbers of participants from 25 to 44 years of age and from 45 to 65 years of age and similar numbers of men and women. Pregnant women and adults unable to understand the questionnaire were excluded. In Seychelles, participants were selected from an electronic register of all inhabitants living around Victoria while ensuring similar numbers of participants 25–44 years of age and 45–65 years of age and similar numbers of men and women.

Survey officers administered a structured closed-ended questionnaire and performed anthropometric measurements. In view of the lack of a standardized dietary questionnaire in SSA and the large variety of diets across countries in the region, a questionnaire was developed during a two-day meeting with the main investigators of each country with several questions being adapted from World Health Organization (WHO) instruments [17]. Questions assessed household characteristics, socio-demographic characteristics, health-related behaviors, and frequency of selected common food items including food items rich in salt (e.g., processed meats, cheeses, pizzas, savory snacks, bread, soups, and relevant local dishes). The questionnaire also included questions on KAP related to salt intake. Height, weight, and three blood pressure readings were measured. Informed consent was obtained from each participant. In each country, the study was approved by the locally relevant institutional ethical review boards.

Weight was measured with an electronic weighing scale to the nearest 0.1 kg. Height was measured with a fixed height rod to the nearest 0.1 cm in all countries. Overweight and obesity were defined for body mass index (BMI) between 25 and 29 or ≥30 kg/m^2^, respectively. Blood pressure was measured with an electronic blood pressure device and high blood pressure defined as systolic/diastolic blood pressure ≥140/90 mmHg or taking treatment for hypertension.

We assessed associations between KAP variables and selected predictors using Spearman correlation coefficients and using stratified analysis. We used the chi-square test to test for differences between categories. Means for all countries were weighted so that each country had the same weight. The level of significance was set at 0.05. Analyses were conducted using Stata 14.1 (StataCorp, College Station, TX, USA).

Publication of data on KAP from this study was delayed because the assessment of salt excretion, which was originally another goal of this study in addition to KAP, had to be cancelled due to funding and other issues. Moreover, sample size was limited when compared to the initially four-time larger anticipated sample size due to discontinued funding by the donor.

## 3. Results

### 3.1. Sample Characteristics

A total of 588 adults between 25 and 65 years old participated in the survey. The characteristics of the participants and their households are described in Table 1. The country samples included similar proportions of men versus women and younger persons (25–44 years of age) versus older person (45–65 years of age). This was consistent with the selection strategy of the participants. Slightly more than half of the participants (54%) were overweight or obese and approximately one-fourth (26%) had high blood pressure. Nearly all households had electricity (90%), a television set (87%), or a radio (89%). Women cooked food more often than men with 60% of the women cooking food every day versus 36% of the men. In addition, 5.4% of the women never cooked versus 20% of the men.

### 3.2. Food Consumption

The usual consumption of foods in each country is shown in Table 2. The most frequently consumed staple foods were maize in Benin, rice and bread in Guinea, Mozambique, and Seychelles, and maize and bread in Kenya. Fish was the most frequent source of animal protein in all countries. The most frequently consumed beverages were tea in Kenya, Mozambique, and Seychelles, coffee in Guinea, and fruit juices in Benin. Participants consumed on average 2.7 meals (95% confidence interval (CI) 2.6–2.7) and 1.2 snacks (95% CI 1.2–1.3) per day and ate 3.0 meals (95% CI 2.8–3.3) outside the home on a weekly basis.

A number of processed food items with known high salt content including processed meat, cheese, pizzas, breakfast cereals, and savory snacks were consumed rather infrequently in all the countries. However, soup and bread, which often contain high amounts of salt, were consumed frequently in all countries. As mentioned in the next paragraph, salt-rich condiments (e.g., Maggi cubes and food spreads such as Marmite/Vegemite), which are often added in soups and other dishes, were also used frequently in all countries.

### 3.3. Knowledge, Attitudes, and Practices (KAP) Related to Salt

Levels of KAP related to salt intake are shown in Table 3. The majority of the participants knew that high salt intake can cause health problems (85%) and could name at least one adequate health problem that can arise from high salt intake (66%). Most of the participants thought it was important to limit salt intake (91% ‘very important’ and ‘somehow important’). However, only 56% of the respondents often tried to limit their salt intake. Moreover, only a small proportion of respondents thought that they consumed too much salt (8%), while a substantial proportion of the respondents thought they consumed too little salt (26%).

Most participants reported that salt was added to the foods most of the time during cooking (92% ‘often’ and ‘always’). Salty condiments such as bouillon cubes, aroma enhancing powders, and sauces were used frequently in all countries especially in Benin, Guinea, and Mozambique (87%, 61%, and 72% added ‘often’ and ‘always’). In contrast, few participants reported adding salt to meals at the table (11% ‘often’ and ‘always’).

### 3.4. Associations between KAP Related to Salt and Socio-Demographic Characteristics

The distribution of several KAP variables was significantly different between countries (*p* < 0.001), but not consistent with sex, age, education, and hypertension treatment (see Supplemental Table S1). Women added salt and other salty condiments during cooking more often than men (*p* = 0.045 and *p* = 0.006, respectively) and added salt less often at the table (*p* = 0.043). KAP levels did not differ significantly between participants who completed primary school vs. those who did not, except for the use of salty condiments (such as bouillon cubes), which were used more frequently among persons with lower education levels (*p* < 0.001). KAP levels did not differ according to anti-hypertensive treatment, except that salty condiments were used less frequently by treated persons (*p* < 0.05) and treated persons thought more often that they consumed too little salt (*p* < 0.05).

Most of the KAP variables were not associated with each other (data not shown). The small sample sizes in several categories precluded meaningful statistical analyses. However, lower discretionary use of salt was associated with higher levels of knowledge related to salt intake (“thinks high salt intake can cause serious health problems”: ρ = −0.23, *p* < 0.001, “knows at least one correct salt-related health problem”: ρ = −0.09, *p* < 0.05, “thinks it is important to limit salt intake”: ρ = −0.20, *p* < 0.001).

## 4. Discussion

Overall, the study shows that the distribution of intake of food items varied widely between countries. Several processed food items with known or presumably high salt content (such as pizzas and savory snacks) were consumed rather infrequently (<3 times/week), but soups and bread were consumed frequently in all countries (>3 times/week), which may suggest substantial salt intake. The study also shows a fairly good level of KAP in relation to salt intake in urban settings among five countries in Africa. However, there were some gaps. A fairly modest proportion of persons added salt at the table. The large majority of participants were aware of health risks related to salt intake and recognized the importance of limiting dietary salt intake. Yet, less than one in ten participants believed they consumed too much salt. We did not find substantial associations within KAP variables or between KAP variables and socio-demographic characteristics, except for an inverse association between the knowledge of the need to restrict salt intake and the discretionary use of salt at the table.

Food consumption differed largely between countries, which underlies the difficulty of developing dietary questionnaires and nutritional guidelines that could apply to all countries. However, certain processed foods such as soups and bread were consumed frequently in all countries. These findings suggest the need for voluntary or mandatory reformulation strategies to reduce the salt content of selected manufactured foods that are both commonly consumed and have high salt content. A study conducted in the early 2000s in South Africa reported that bread was a main source of dietary salt in this country [18]. As a result, the South African government regulated the maximum levels of salt permitted in a wide range of industrially processed food categories, including breads, in order to reduce salt intake in the population [19,20]. Reformulation policies aimed at reducing salt in manufactured foods can be highly cost-effective [21] and are recommended by the WHO’s Global Action Plan for the Prevention and Control of Non-Communicable Diseases [4]. Several countries have implemented policies to reformulate selected manufactured foods with subsequent reductions of the salt intake at the population level [22,23,24]. However, none of the five countries included in this survey have implemented such reformulation strategies [9]. South Africa is the only country in the African region that has taken regulatory steps to mandatorily reduce the salt content of selected foods [9]. Findings of our study also emphasize the need for continued education campaigns in order to encourage people to limit dietary salt intake. Such campaigns are also useful when advocating for studies assessing sources of salt intake in a particular population and when advocating for corresponding reformulation strategies. Policy aimed at reformulating foods frequently consumed and high in salt is a cornerstone strategy for effective reduction of salt intake in the population.

Knowledge on the detrimental effect of high salt intake was fairly high and higher than could have been anticipated from previous research [10,11,12,13,14]. However, fairly good knowledge about health effects of salt did not seem to have translated into strong attitudes and practices with regards to salt intake reduction. For example, knowledge that salt could be detrimental for health was associated with only one salt-related practice, i.e., low discretionary use of salt. A study conducted in 2014–2015 in Mozambique also found participants high in knowledge but low in attitudes and practices, in which is similar to our study [25]. This suggests that campaigns aimed at raising awareness about the detrimental impact of salt intake on health might better translate in actual salt reduction if structural measures are also implemented, e.g., programs to reduce salt in the food served in work or school canteens and measures to limit salt intake in selected manufactured foods (e.g., bread). This is consistent with a review showing that the implementation of education and awareness-raising interventions alone is unlikely to be adequate in reducing population salt intake to the recommended levels, which suggests that behavior change might better occur when combining health education and public awareness campaigns [26]. The quasi-ubiquitous presence of television and radio in the surveyed households suggests that these media could be the main instruments to relay such public awareness campaigns.

The main strengths of this study were the inclusion of population-based samples in five countries and the use of the same methodology, which allows direct comparison between countries. The study also has limitations. First, salt intake was not measured in all countries and we cannot report on the relation between salt-related behaviors and an objective measurement of salt intake. Moreover, our study did not allow for the quantification of salt added at the table or the contribution of salt in the form of processed versus non-processed foods. Second, a number of selection biases may have occurred in the sampling of participants. For example, persons present in a household at the time of the survey may have been different than persons absent. This may, however, have limited impact, since food consumption tends to be fairly homogenous at a household level. Individuals from a low socioeconomic status (e.g., the illiterate persons unable to understand questions) may also have been under-represented in this study. However, this proportion is likely small and has little overall impact on the results. Our survey was limited to urban areas and the generalization of the findings is, therefore, limited to such urban areas. Admittedly, food consumption may differ largely in rural areas and further studies need to be conducted in these different areas. Yet, urbanization is rapidly increasing in SSA and findings in this study may reflect dietary habits among large segments of the population on the continent. Third, the study relied on reported information, which is prone to recall and other biases that can lead to under-reporting or over-reporting of certain foods and practices. Fourth, the fairly low numbers of participants precluded meaningful statistical analyses of associations between KAP variables and socio-demographic characteristics.

Very little data is available on actual salt intake, the sources of salt intake, and KAP related to salt in SSA. One systematic review aimed to identify all published studies reporting salt intake in countries of SSA until 2015 [27]. This review found that 81% of the adult populations consumed salt intake above the recommended maximum 5 g per day [27], which suggests overall high intake in the region. Intake was higher in urban than in rural populations [27]. With regard to countries included in this study, the review identified one study in children in Benin in 1996 [28] and one study in Kenya in 1986 in rural areas [29]. When looking for more recent studies, urinary excretion of salt was assessed in Benin based on the same study on KAP and in Mozambique using another study. Both studies found high salt intake (10.2 g and 10.5 g of salt per day in Benin and Mozambique, respectively) [30,31]. A global modelling study [32] estimated that mean salt intake in all of the five countries in our study, apart from Kenya, was above the maximum 5 g of salt recommend by the WHO [33].

Future research on KAP related to salt is recommended in these countries among others in the region. An objective measurement of the actual amount of salt consumed in these populations is needed to assess whether salt intake is above recommendations and in which population groups. In addition, further qualitative studies should examine specific practices that may favor salt intake. Lastly, analyses of salt intake of foods especially in bread, instant soups and selected local foods as well as market studies are needed to identify the main sources of dietary salt in these populations in order to guide reformulation guidelines.

## 5. Conclusions

In conclusion, our study among adults in urban settings found largely different food consumption patterns between countries but consumption of salt-rich bread, soup, and salty condiments was frequent in all countries, which suggests that the salt intake could be substantial in all countries. We found fairly good knowledge related to the detrimental effects of high salt intake, but mixed findings related to the attitudes and practices related to the reduction of salt intake. These findings support the need for both education campaigns to promote knowledge, attitudes, and behaviors for the control of dietary salt intake and reformulation strategies to reduce salt content of selected frequently eaten foods high in salt.

## Figures and Tables

**Table 1:** Socio-demographic characteristics of the participants ^1^.

Characteristics	All ^2^	Benin	Guinea	Kenya	Mozambique	Seychelles
*Participant characteristics*						
Total sample size (*n*)	588	140	119	102	77	150
Female (%)	54 (50−58)	51 (43–60)	52 (43–61)	49 (39–59)	61 (50–71)	58 (50–66)
Age (mean)	42 (41–43)	42 (41–44)	43 (41–45)	43 (41–45)	41 (39–43)	42 (40–44)
25−44 years (%)	57 (53−61)	54 (45–62)	59 (50–67)	54 (44–63)	61 (50–71)	56 (48–64)
45−65 years (%)	43 (39–47)	46 (38–55)	41 (33–50)	46 (37–56)	39 (29–50)	44 (36–52)
Completed primary school (%)	80 (76–83)	56 (47–64)	78 (70–85)	88 (80–93)	77 (66–85)	99 (95–100)
Overweight (%)	30 (26–34)	31 (24–39)	24 (17–32)	24 (16–33)	32 (22–43)	40 (32–48)
Obese (%)	24 (21–28)	24 (17–31)	16 (10–24)	23 (16–32)	29 (20–41)	29 (23–37)
High blood pressure or treatment for hypertension (%)	26 (22–30)	26 (20–34)	18 (12–26)	25 (18–35)	26 (17–37)	37 (29–45)
Treatment for hypertension (%)	15 (12–18)	9 (5–15)	13 (8–21)	17 (11–25)	17 (10–27)	19 (13–26)
*Household assets*						
Running water (%)	74 (70–78)	68 (60–75)	83 (75–89)	30 (22–40)	90 (80–95)	99 (95–100)
Electricity (%)	90 (87–92)	86 (80–91)	99 (94–100)	70 (60–78)	96 (89–99)	100 (100–100)
Fridge (%)	59 (55–63)	19 (13–26)	71 (62–78)	30 (22–40)	77 (66–85)	100 (100–100)
Radio (%)	89 (86–92)	87 (80–92)	80 (72–87)	86 (78–92)	94 (85–97)	99 (95–100)
Television (%)	87 (84–89)	77 (69–83)	92 (86–96)	66 (56–74)	99 (91–100)	100 (100–100)
Cable television (%)	47 (43–51)	48 (40–56)	79 (71–85)	29 (21–39)	16 (9–26)	65 (57–72)
Bicycle or motorcycle (%)	28 (25–32)	64 (55–71)	29 (21–37)	30 (22–40)	5 (2–13)	13 (8–19)
Car or truck (%)	24 (21–28)	6 (3–12)	40 (32–49)	8 (4–15)	22 (14–33)	43 (36–51)

^1^ Values are means (95% confidence intervals). ^2^ Estimates for all were weighted so that data from each country had the same weight.

**Table 2:** Consumption of selected food items according to country ^1^.

Food item	Benin	Guinea	Kenya	Mozambique	Seychelles
*Staple foods*					
Rice					
Maize					
Potato					
Yam					
Bread					
*Fruits and vegetables*					
Salad					
Vegetables					
Fruits					
*Animal products*					
Chicken					
Red meat					
Processed meat					
Fish					
Eggs					
Cheese					
*Other foods*					
Soup					
Pizza					
Breakfast cereals					
Savory snacks					
Sweets and pastries					
Supplements or vitamins					
*Non-alcoholic beverages*					
Tea					
Coffee					
Commercial soft drink					
Locally made lemonade					
Fresh fruit juice					
Non-fresh fruit juice					
Milk					

^1^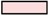: <1 times/week, 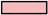: 1–3 times/week, 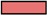: 4–6 times/week, 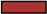: every day.

**Table 3:** Knowledge, attitudes, and practices related to salt intake according to country (in %).

Knowledge, Attitudes, and Practices	All ^1^	Benin	Guinea	Kenya	Mozambique	Seychelles
**Knowledge**						
*High salt intake can cause serious health problems*						
Yes	85 (82–88)	93 (88–97)	66 (57–74)	87 (79–92)	88 (78–93)	92 (86–95)
No	5 (3–7)	2 (1–7)	5 (2–11)	6 (3–13)	4 (1–12)	6 (3–11)
Don’t know	10 (8–13)	4 (2–9)	28 (21–37)	7 (3–14)	8 (4–17)	2 (1–6)
*Health problems are associated with high salt intake*						
≥1 problem known	66 (61–70)	87 (78–92)	64 (52–74)	48 (38–59)	93 (81–98)	50 (41–59)
None known	34 (30–39)	13 (8–22)	36 (26–48)	52 (41–62)	7 (2–19)	50 (41–59)
*It is important to limit salt intake*						
Very important	9 (7–12)	7 (4–13)	20 (13–28)	7 (3–14)	7 (3–15)	5 (2–10)
Somehow important	12 (9–14)	4 (1–8)	23 (16–32)	14 (9–23)	0 (0–0)	17 (12–24)
Not really important	79 (76–82)	89 (83–93)	57 (48–66)	79 (70–86)	93 (85–97)	78 (70–84)
**Attitudes**						
*Try to limit salt*						
Often	55 (51–59)	76 (68–82)	47 (38–57)	54 (44–63)	66 (54–76)	32 (25–40)
Sometimes	16 (13–19)	11 (7–18)	18 (12–26)	26 (18–36)	3 (1–11)	22 (16–29)
Not really	29 (25–33)	13 (8–20)	35 (26–44)	20 (13–29)	31 (21–43)	46 (38–54)
*Perceived amount of salt consumed*						
Too little	26 (22–30)	2 (1–6)	55 (46–64)	36 (27–45)	30 (21–41)	7 (4–12)
About right	59 (55–63)	96 (92–99)	20 (14–28)	52 (43–62)	60 (48–70)	67 (59–74)
Too much	8 (6–10)	1 (0–5)	5 (2–11)	12 (7–20)	10 (5–20)	11 (7–17)
Don’t know	7 (5–9)	1 (0–5)	19 (13–27)	0 (0–0)	0 (0–0)	16 (11–23)
**Practices**						
*Salt is added during cooking*						
Never	2 (1–3)	2 (1–6)	3 (1–8)	2 (0–8)	0 (0–0)	1 (0–5)
Sometimes (1–2 times/week)	7 (5–9)	1 (0–5)	27 (20–36)	3 (1–9)	3 (1–10)	0 (0–0)
Often (most meals)	19 (16–22)	5 (2–10)	18 (12–26)	30 (22–39)	27 (18–38)	15 (10–21)
Always (all meals)	73 (69–76)	92 (86–96)	52 (43–61)	65 (56–74)	70 (59–79)	84 (77–89)
*Salty condiments are used during cooking ^2^*						
Never	9 (6–11)	6 (3–12)	8 (4–14)	21 (14–31)	4 (1–11)	4 (2–9)
Sometimes (1–2 times/week)	26 (23–30)	7 (4–13)	29 (22–38)	32 (23–42)	25 (16–36)	38 (30–46)
Often (most meals)	22 (18–25)	9 (5–15)	11 (6–18)	24 (17–34)	25 (16–36)	40 (32–48)
Always (all meals)	42 (38–47)	78 (70–84)	50 (41–59)	17 (11–26)	47 (36–58)	18 (13–25)
*Salt is added to food at the table*						
Never	66 (62–70)	71 (63–78)	46 (37–55)	46 (36–56)	82 (72–89)	87 (80–91)
Sometimes (1–2 times/week)	23 (20–27)	24 (17–31)	33 (25–42)	37 (28–47)	12 (6–21)	9 (6–15)
Often (most meals)	6 (4–8)	2 (1–6)	14 (8–21)	8 (4–15)	4 (1–11)	2 (1–6)
Always (all meals)	5 (3–7)	3 (1–7)	8 (4–14)	9 (5–16)	3 (1–10)	2 (1–6)
*Consumption of foods high in salt ^3^*						
Never	34 (30–38)	1 (0–6)	41 (32–50)	16 (10–24)	83 (73–90)	28 (21–36)
1–2 times/week	15 (13–18)	7 (4–13)	38 (30–48)	18 (11–27)	1 (0–9)	12 (8–18)
3–4 times/week	29 (25–32)	5 (2–10)	9 (5–16)	54 (45–64)	16 (9–26)	58 (50–66)
Every day/almost every day	21 (18–25)	86 (79–91)	6 (3–12)	12 (7–20)	0 (0–0)	2 (1–6)

^1^ Estimates for all were weighted so that data from each country had the same weight. ^2^ These condiments included bouillon cubes, Aromat powder, soy sauce, food spreads (e.g., Vegemite, Marmite), and similar items. ^3^ These foods included salted fish, salted meat, salami, salted peanuts, food spreads, pizza, and other typical local meals rich in salt.

## Supplementary Materials

The following are available online at http://www.mdpi.com/2072-6643/10/8/1028/s1, Table S1: Knowledge, attitudes, and practices related to salt intake according to sex, age, education, and hypertension treatment.
