# Supplementary material for: Food Consumption, Knowledge, Attitudes, and Practices Related to Salt in Urban Areas in Five Sub-Saharan African Countries
Source: Nutrients. 2018 Aug 7;10(8):1028. doi: 10.3390/nu10081028 (PMC6116014; doi:10.3390/nu10081028)
Supplement: Supplementary file 1 [file nutrients-10-01028-s001.pdf]

**Table S1.** Knowledge, attitudes and practices related to salt intake according to sex, age, education and hypertension treatment (in %).

| | Male | Female | 24–44 years | 45–65 years | With primary education | Without primary education | Hypertension treatment | No treatment |
|---|---|---|---|---|---|---|---|---|
| **Knowledge** | | | | | | | | |
| *High salt intake can cause serious health problems* | | | | | | | | |
| Yes | 83 (78–87) | 88 (84–91) | 85 (81–89) | 87 (82–90) | 85 (82–88) | 88 (81–93) | 93 (85–97) | 88 (84–90) |
| No | 6 (3–9) | 4 (2–7) | 6 (4–9) | 4 (2–7) | 5 (3–7) | 3 (1–9) | 1 (0–8) | 4 (3–7) |
| Don’t know | 11 (8–2) | 8 (6–12) | 9 (7–13) | 10 (7–14) | 10 (7–13) | 8 (5–15) | 6 (2–13) | 8 (6–11) |
| *Health problems are associated with high salt intake* | | | | | | | | |
| ≥1 problem known | 65 (58–72) | 63 (57–69) | 61 (55–67) | 68 (61–74) | 62 (57–67) | 76 (64–84) | 65 (64–75) | 63 (58–68) |
| None known | 35 (28–42) | 37 (31–43) | 39 (33–45) | 32 (26–39) | 38 (33–43) | 24 (16–36) | 35 (25–46) | 37 (32–429 |
| *It is important to limit salt intake* | | | | | | | | |
| Very important | 10 (7–14) | 8 (5–11) | 10 (8–14) | 7 (4–11) | 9 (7–12) | 8 (4–14) | 6 (2–13) | 9 (7–12) |
| Somehow important | 15 (11–20) | 10 (8–14) | 14 (11–18) | 10 (7–15) | 13 (11–17) | 9 (5–15) | 10 (6–19) | 12 (9–15) |
| Not really important | 75 (70–80) | 82 (77–86) | 75 (70–80) | 83 (78–87) | 8 (1–8) | 8 (8–9) | 84 (74–90) | 79 (75–83) |
| **Attitudes** | | | | | | | | |
| *Try to limit salt* | | | | | | | | |
| Often | 20 (16–25) | 26 (22–32) | 21 (17–25) | 27 (22–33) | 23 (19–27) | 25 (18–34) | 39 (29–50) | 17 (14–21) |
| Sometimes | 62 (56–68) | 60 (54–65) | 64 (59–69) | 57 (51–63) | 60 (55–64) | 66 (57–74) | 44 (34–54) | 68 (63–72) |
| Not really | 11 (76–15) | 4 (3–7) | 7 (5–11) | 7 (5–11) | 8 (6–11) | 4 (2–10) | 7 (3–15) | 8 (6–11) |
| *Perceived amount of salt consumed* | | | | | | | | |
| Too little | 46 (40–52) | 61 (55–66) | 50 (44–55) | 59 (53–65) | 50 (45–54) | 70 (61–78) | 65 (54–75) | 56 (52–61) |
| About right | 20 (15–25) | 14 (11–19) | 17 (13–21) | 17 (13–22) | 19 (16–23) | 8 (4–14) | 16 (93–25) | 18 (15–22) |
| Too much | 34 (29–40) | 25 (20–30) | 33 (28–39) | 24 (19–30) | 31 (27–36) | 22 (15–30) | 19 (12–29) | 26 (22–30) |
| Don’t know | 46 (40–52) | 61 (55–66) | 50 (44–55) | 59 (53–65) | 50 (45–54) | 70 (61–78) | 65 (54–75) | 56 (52–61) |
| **Practices** | | | | | | | | |
| *Salt is added during cooking* | | | | | | | | |
| Never | 1 (0–3) | 2 (1–5) | 2 (0–4) | 2 (0–5) | 2 (0–3) | 2 (0–7) | 2 (0–9) | 2 (0–3) |
| Sometimes (1–2 times/week) | 9 (6–13) | 4 (2–7) | 8 (5–11) | 5 (3–9) | 7 (5–10) | 3 (1–9) | 3 (1–10) | 4 (3–7) |
| Often (most meals) | 19 (14–24) | 16 (12–21) | 17 (14–22) | 17 (13–22) | 18 (15–22) | 13 (8–21) | 21 (13–31) | 15 (12–19) |
| Always (all meals) | 71 (65–76) | 78 (73–82) | 74 (69–78) | 75 (70–80) | 73 (69–77) | 81 (73–87) | 72 (62–81) | 79 (75–82) |
| *Salty condiments are used during cooking*[1] | | | | | | | | |
| Never | 11 (8–15) | 6 (4–9) | 7 (4–10) | 10 (7–14) | 9 (7–12) | 6 (3–12) | 13 (7–21) | 8 (6–11) |
| Sometimes (1–2 times/week) | 25 (20–30) | 27 (22–32) | 24 (20–29) | 28 (23–34) | 28 (25–33) | 16 (10–24) | 34 (25–45) | 21 (17–25) |
| Often (most meals) | 21 (16–26) | 23 (18–28) | 23 (19–28) | 20 (15–25) | 26 (22–30) | 7 (3–13) | 20 (12–29) | 21 (18–25) |
| Always (all meals) | 41 (35–47) | 44 (39–50) | 44 (39–50) | 41 (35–47) | 35 (31–40) | 71 (63–79) | 32 (23–43) | 49 (44–53) |
| *Salt is added to food at the table* | | | | | | | | |
| Never | 62 (56–68) | 72 (66–76) | 67 (61–72) | 68 (62–73) | 67 (62–71) | 69 (60–77) | 80 (71–88) | 67 (62–71) |

| | | | | | | | | |
|---|---|---|---|---|---|---|---|---|
| Sometimes (1–2 times/week) | 28 (23–33) | 18 (14–23) | 23 (19–28) | 22 (17–27) | 23 (20–27) | 19 (13–27) | 13 (7–21) | 23 (19–27) |
| Often (most meals) | 5 (3–9) | 6 (4–9) | 5 (3–9) | 6 (4–10) | 5 (4–8) | 7 (3–13) | 2 (0–9) | 5 (4–8) |
| Always (all meals) | 5 (3–9) | 4 (2–7) | 5 (3–8) | 4 (2–8) | 5 (3–7) | 5 (2–11) | 5 (2–12) | 5 (3–8) |
| *Consumption of foods high in salt*[2] | | | | | | | | |
| Never | 25 (20–30) | 33 (28–39) | 30 (25–35) | 29 (24–35) | 29 (25–33) | 30 (22–39) | 37 (27–47) | 29 (25–33) |
| 1–2 times/week | 17 (13–22) | 15 (11–19) | 17 (13–22) | 14 (10–19) | 18 (15–22) | 7 (3–13) | 10 (5–19) | 13 (10–16) |
| 3–4 times/week | 30 (25–36) | 29 (24–34) | 29 (24–34) | 30 (25–36) | 34 (30–39) | 10 (6–17) | 37 (27–47) | 28 (24–33) |
| Every day or almost every day | 26 (21–32) | 23 (18–28) | 23 (19–28) | 26 (21–32) | 17 (14–21) | 51 (42.60) | 16 (10–25) | 29 (25–34) |

[1] These condiments included bouillon cubes, Aromat powder, soy sauce, food spreads (e.g., Vegemite, Marmite), and similar items; [2] These foods included salted fish, salted meat, salami, salted peanuts, food spreads, pizza, and other typical local meals rich in salt.
